# Probabilistic models for neural populations that naturally capture global coupling and criticality

**DOI:** 10.1371/journal.pcbi.1005763

**Published:** 2017-09-19

**Authors:** Jan Humplik, Gašper Tkačik

**Affiliations:** Institute of Science and Technology Austria, Klosterneuburg, Austria; University of Edinburgh, UNITED KINGDOM

## Abstract

Advances in multi-unit recordings pave the way for statistical modeling of activity patterns in large neural populations. Recent studies have shown that the summed activity of all neurons strongly shapes the population response. A separate recent finding has been that neural populations also exhibit criticality, an anomalously large dynamic range for the probabilities of different population activity patterns. Motivated by these two observations, we introduce a class of probabilistic models which takes into account the prior knowledge that the neural population could be globally coupled and close to critical. These models consist of an energy function which parametrizes interactions between small groups of neurons, and an arbitrary positive, strictly increasing, and twice differentiable function which maps the energy of a population pattern to its probability. We show that: 1) augmenting a pairwise Ising model with a nonlinearity yields an accurate description of the activity of retinal ganglion cells which outperforms previous models based on the summed activity of neurons; 2) prior knowledge that the population is critical translates to prior expectations about the shape of the nonlinearity; 3) the nonlinearity admits an interpretation in terms of a continuous latent variable globally coupling the system whose distribution we can infer from data. Our method is independent of the underlying system’s state space; hence, it can be applied to other systems such as natural scenes or amino acid sequences of proteins which are also known to exhibit criticality.

## Author summary

Populations of sensory neurons represent information about the outside environment in a collective fashion. A salient property of this distributed neural code is criticality. Yet most models used to date to analyze recordings from large neural populations do not take this observation explicitly into account. Here we aim to bridge this gap by designing probabilistic models whose structure reflects the expectation that the population is close to critical. We show that such principled approach improves previously considered models, and we demonstrate a connection between our models and the presence of continuous latent variables which is a recently proposed mechanism underlying criticality in many natural systems.

## Introduction

Recent progress in recording technology that permits monitoring the activity of large neural populations simultaneously has enabled us to infer detailed large-scale probabilistic models for neural activity and, hence, to document and interpret patterns of statistical dependencies between neural responses. Many questions regarding collective behavior in large populations of sensory neurons, previously in the domain of theoretical speculation, were thus brought into the spotlight and into contact with rich experimental data: How can large populations of sensory neurons encode information reliably despite the noise, and how can the stimulus information be recovered? How can downstream areas “learn” to read the neural code without direct access to the stimulus? Are there statistical features of the neural code that point to “design principles” at the population level and provide a prior over the space of possible neural codes? While stimulus-conditional (encoding) [1–3] and decoding approaches [4–6] have been instrumental for understanding stimulus representation, probabilistic models for the total distribution of neural activity [7] highlighted various salient statistical features of the neural code, two of which we focus on below.

The first salient feature is that neural populations are often “globally coupled.” While it has been appreciated for some time that neurons do not spike independently, the approximation that their interactions are well-described by low-order statistical dependencies (e.g., pairwise interactions) has provided powerful descriptions of the data, known as pairwise maximum entropy (Ising-like) models or, alternatively, as fully-visible Boltzmann machines [8–10]. As the recorded populations grew to tens or hundreds of neurons, however, it became increasingly clear that pairwise models are insufficient [11]. Instead of increasing model complexity order-by-order (e.g., by including triplet interactions [12]) which quickly becomes intractable, one proposal has been to directly identify global or collective modes of activity and build models that reproduce them well. In the retina, for example, the population synchrony, or the summed activity over all neurons in a given time bin, represents one such global statistic that probabilistic models can reproduce, leading to the so-called “K-pairwise” models [11, 13, 14]. Similar ideas relate to models able to capture the no-spike probability in groups of neurons in hippocampal slices [15], or correlation between population synchrony and firing of individual neurons in the cortex [16–18]. In all cases, the increased performance of the proposed models originates in the models’ ability to capture higher-order correlations in neural spiking through a smart guess for the global (macroscopic) statistic of the population activity.

The second salient feature is that neural population responses are close to critical in a thermodynamic sense [10, 19]. This criticality is distinct from the dynamical, avalanche-type criticality that has been studied extensively in the past [20, 21], although formal connections between the two notions may exist [22]. We give a precise definition of thermodynamic criticality below. Intuitively and informally, criticality of the ensemble of patterns of spikes and silences implies the following: (i) the distribution of neural responses is Zipfian, with a slope of −1 on a log-frequency vs log-rank plot; equivalently, the (log) density of states and (log) probability of responses are linearly related [19, 23]; (ii) the dynamic range of neural response probabilities is anomalously large in a certain mathematical sense [10]; (iii) there is no clearly definable information-theoretic “typical set” of responses; (iv) even though responses are of high dimensionality, one is likely to observe certain patterns of spiking and silence multiple times in a typical experiment [14, 24].

Several works pursued the origins of the observed signatures of criticality [25–30]. Two recent papers [28, 29] focused on the role of unobserved (latent) variables whose fluctuations, coupled to the observable responses of individual neurons, could lead to critical response ensemble under relatively generic conditions. While these works provided an interesting proof-of-concept analysis, it has remained unclear whether these ideas could be incorporated into a probabilistic model that could be tractably inferred from large-scale data and that would simultaneously recapitulate the critical behavior through the proposed mechanism, match in detail the many previously documented statistical features of the neural code, and provide cross-validated performance similar to existing state-of-the-art models.

Here we introduce a new class of probabilistic models for the neural code called *semiparametric energy-based models*. These models explicitly incorporate our prior belief that the neural population could be globally coupled and close to critical. If data indeed exhibits such features, our models can capture them efficiently; otherwise, our models can reduce to previously studied energy-based (e.g., pairwise maximum entropy) models. We infer our models on populations of 100+ retinal ganglion cells and show that they provide superior performance over K-pairwise models. We further show that our models capture critical behavior by a mechanism that is mathematically equivalent to the fluctuating latent variable model, and give an interpretation of the resulting latent variable as defining the state of the retinal population to be “active” or “silent.” Importantly, the central idea of the framework introduced here extends beyond the neural code in general and the retina in particular: any energy-based probabilistic model can be augmented with our proposed mechanism. This flexibility is relevant since other interesting datasets, such as natural image patches [31, 32] or certain genomic sequences [33], also exhibit critical and globally coupled nature.

### Models of globally coupled neural populations

We represent the response of a neural population with a binary vector **s** = {*s*_1_, *s*_2_, …, *s*_*N*_} ∈ {0, 1}^*N*^ identifying which of the *N* neurons elicited at least one action potential (‘1’) and which stayed silent (‘0’) during a short time window. Our goal is to build a model for the probability distribution of activity patterns, *p*(**s**), given a limited number *M* of samples, $D=\{s^{\left(1\right)},\ldots ,s^{\left(M\right)}\}$, observed in a typical recording session. The regime we are mainly interested in is the one where the dimensionality of the problem is sufficiently high that the distribution *p* cannot be directly sampled from data, i.e., when 2^*N*^ ≫ *M*. Note that we are looking to infer models for the unconditional distribution over neural activity patterns (i.e., the population “vocabulary”), explored in a number of recent papers [8, 9, 11, 13–18, 24, 34], rather than to construct stimulus-conditional models (i.e., the “encoding models”, which have a long tradition in computational neuroscience [1–3]).

Previous approaches to modeling globally coupled populations focused on the total network activity, also known as synchrony, $K\left(s\right)=\sum_{i=1}^{N}\;s_{i}$. The importance of this quantity was first analyzed in the context of probabilistic models in Ref [11] where the authors showed that a K-pairwise model, which generalizes a pairwise maximum entropy model by placing constraints on the statistics of *K*(**s**), is much better at explaining the observed population responses of 100+ salamander retinal ganglion cells than a pairwise model. Specifically, a pairwise model assumes that the covariance matrix between single neuron responses, *C*_*ij*_ = 〈*s*_*i*_*s*_*j*_〉, which can be determined empirically from data D, is sufficient to estimate the probability of any population activity pattern. In the maximum entropy framework, this probability is given by the most unstructured (or random) distribution that reproduces exactly the measured *C*_*ij*_:

$$\begin{array}{c} p\left(s;J\right)=\frac{1}{Z(J)}\exp\;(\sum_{i,j=1}^{N}\;J_{ij}s_{i}s_{j}), \end{array}$$ (1)

where *Z*(***J***) is a normalization constant, and ***J*** is a coupling matrix which is chosen so that samples from the model have the same covariance matrix as data. Note that because $s_{i}^{2}=s_{i}$, the diagonal terms *J*_*ii*_ of the coupling matrix correspond to single neuron biases, i.e. firing probabilities in the absence of spikes from other neurons (previous work [11] used a representation *s*_*i*_ ∈ {−1, 1} for which the single neuron biases need to be included as separate parameters and where *J*_*ii*_ are all 0). A K-pairwise model generalizes the pairwise model and has the form

$$\begin{array}{c} p\left(s;J,\phi \right)=\frac{1}{Z(J,\phi )}\exp\;(\sum_{i,j=1}^{N}\;J_{ij}s_{i}s_{j}+\sum_{k=0}^{N}\;\phi_{k}\delta_{k,K(s)}). \end{array}$$ (2)

The coupling matrix ***J*** has the same role as in a pairwise model while the additional parameters *ϕ* are chosen to match the probability distribution of *K*(**s**) under the model to that estimated from data. The “potentials” *ϕ*_*k*_ introduced into the K-pairwise probabilistic model, Eq (2), globally couple the population, and cannot be reduced to low-order interactions between, e.g., pairs or triplets, of neurons, except in very special cases. We will generically refer to probabilistic models that impose non-trivial constraints on population-level statistics (of which the distribution of total network activity *K* is one particular example) as “globally coupled” models.

**Fig 1:**
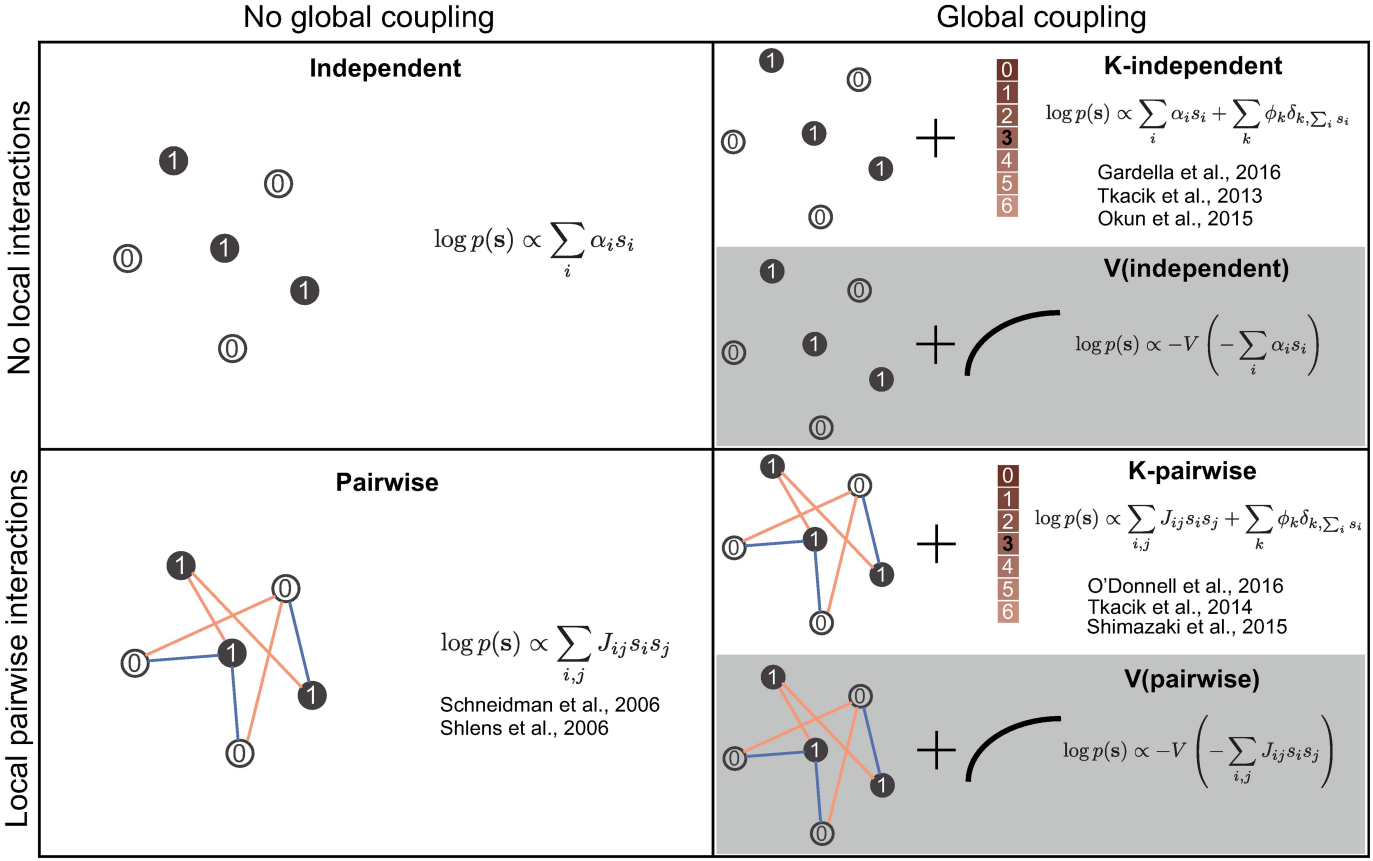
Overview of models which contain mechanisms for capturing global coupling. At any given time, the population activity pattern is defined by neurons which either spike (*s*_*i*_ = 1, dark discs) or are silent (*s*_*i*_ = 0, white discs). The probability of spiking is partially determined by an intrinsic firing bias (*α*_*i*_ for models without local interactions, or the diagonal terms of the coupling matrix *J* for models with local pairwise interactions). When local interactions between neurons are important, they can be parametrized by assigning each pair of neurons a coupling weight. Positive weight (orange) increases the likelihood of the paired neurons spiking together, while negative weight (blue) decreases the likelihood. The negative sum of the intrinsic firing biases of active neurons and the coupling weights of pairs which fire synchronously is referred to as the energy of the population activity pattern. The probability of a given pattern is simply proportional to the exponential of its negative energy. To capture correlations due to global coupling, previous studies considered models which bias the response probabilities with a function of the total network activity (here denoted as *K*, i.e., the sum of the activities of individual neurons). We introduce a different approach (shaded models in the figure) where global coupling is induced by mapping the energy of the activity pattern to its probability with an arbitrary (smooth and increasing) function exp(−*V* (*E*)).

Here we introduce new *semiparametric energy-based models* that extend the notion of global coupling. These models are defined as follows:

$$\begin{array}{c} p\left(s;\alpha ,V\right)=\frac{e^{-V\;(E(s;\alpha ))}}{Z(\alpha ,V)}, \end{array}$$ (3)

where *E*(**s**; ***α***) is some *energy function* parametrized by ***α***, and *V* is an arbitrary increasing differentiable function which we will refer to simply as the “nonlinearity.” The parametrization of the energy function should be chosen so as to reflect local interactions among neurons. Crucially, while it is necessary to choose a specific parametrization of the energy function, we do not make any assumptions on the shape of the nonlinearity—we let the shape be determined nonparametrically from data. Fig 1 schematically displays the relationship between the previously studied probabilistic models of population activity and two semiparametric energy-based models that we focus on in this paper, the *semiparametric independent model* (which we also refer to as “V(independent)”) and the *semiparametric pairwise model* (which we also refer to as “V(pairwise)”).

Our motivation for introducing the global coupling via the nonlinearity *V* traces back to the argument made in Ref [11] for choosing to constrain the statistics of synchrony, *K*(**s**); in short, the key intuition in earlier work has been that *K*(**s**) is a biologically relevant quantity which encodes information about the global state of a population. There are, however, many other quantities whose distributions could contain signatures of global coupling in a population. In particular, while most energy functions—e.g., the pairwise energy function, *E*(**s**; ***J***) = −∑_*i*,*j*_ *J*_*ij*_*s*_*i*_*s*_*j*_—are defined solely in terms of local interactions between small groups of neurons, the statistics of these same energy functions (for instance, their moments) are strongly shaped by global effects. Specifically, we show in Methods that the role of the nonlinearity in Eq (3) is precisely to match the probability density of the energy under the model to that estimated from data. In other words, once any energy function for Eq (3) has been chosen, the nonlinearity *V* will ensure that the distributions of that particular energy in the model and over data samples agree.

Constraining the statistics of the energy *E*(**s**; ***α***) is different from constraining the statistics of *K*(**s**), used in previous work. First, the energy depends on a priori unknown parameters ***α*** which must be learned from data. Second, while *K*(**s**) is always an integer between 0 and *N*, the energy can take up to 2^*N*^ distinct values; this allows for extra richness but also requires us to constrain the (smoothed) histogram of energy rather than the probability of every possible energy value, to prevent overfitting.

As we discuss next, the statistics of the energy are also closely related to criticality, a formal, model-free property distinguishing large, globally-coupled neural populations.

### Criticality

The notion of criticality originates in thermodynamics where it encompasses several different properties of systems undergoing a second-order phase transition [35]. Today, many other phenomena, such as power-law distributed sizes of “avalanches” in neural activity, have been termed critical [20]. Our definition, which we discuss below, is a restricted version of the thermodynamic criticality.

We consider a sequence of probability distributions ${\left\{p_{N}\right\}}_{N=1}^{\infty }$ over the responses of neural populations of increasing sizes, *N*. These probability distributions define the discrete random variable **s** (the population response), but they can also be thought of simply as functions which map a population response to a number between 0 and 1. Combining these two viewpoints, we can consider a real-valued random variable *p*_*N*_(**s**) ∈ (0, 1) which is constructed by applying the function *p*_*N*_ to the random variable **s**. The behavior of this random variable as *N* → ∞ is often universal, meaning that some of its features are independent of the precise form of *p*_*N*_. As is conventional, we work with the logarithm of *p*_*N*_(**s**) instead of the actual distribution. We call a population “critical” if the standard deviation of the random variable log *p*_*N*_(**s**)/*N* does not vanish as the population size becomes large, i.e.

$$\begin{array}{c} \frac{1}{N}\sigma \;(\log p_{N}\left(s\right))↛\;0\;\text{as}\;N\to \infty . \end{array}$$ (4)

(For completeness, we further exclude some degenerate cases such as when the probability density of log *p*_*N*_(**s**)/*N* converges to two equally sized delta functions.)

The above definition is related to criticality as studied in statistical physics. In thermodynamics, $\sigma \;(\log p_{N}\left(s\right))/\sqrt{N}$ is proportional to the square root of the specific heat, which diverges in systems undergoing a second-order phase transition. While at a thermodynamical critical point *σ* (log *p*_*N*_(**s**))/*N* scales as *N*^−*γ*^ with *γ* ∈ (0, 1/2), here we are concerned with the extreme case of *γ* = 0. Rather than being related to second-order phase transitions, this definition of criticality is related to the so-called Zipf law [23].

A pattern **s** can be assigned a rank by counting how many other patterns have a higher probability. In its original form, a probability distribution is said to satisfy Zipf law if the probability of a pattern is inversely proportional to its rank. No real probability distribution is actually expected to satisfy this definition precisely, but there is a weaker form of Zipf law which concerns very large populations, and which is much less restrictive. This weaker form can be stated as a smoothed version of the original Zipf law. Consider patterns whose rank is in some small interval [*r*, *r* + Δ_*N*_], and denote *p*_*N*_(*r*) the average probability of these patterns. We generalize the notion of Zipf law to mean that for very large populations *p*_*N*_(*r*) ∝ *r*^−1^ (Δ_*N*_ is assumed to go to zero sufficiently quickly with *N*). As shown in Ref [23], a system is critical in the sense of Eq (4) precisely when it follows this generalized Zipf law. Practically speaking, no experimentally studied population ever has an infinite size, and a typical way to check for signs of criticality is to see if a log-log plot of a pattern probability versus its rank resembles a straight line with slope −1.

Most systems are not expected to be critical. The simplest example is a population of identical and independent neurons,

$$\begin{array}{c} p_{N}\left(s\right)=q^{\sum_{i=1}^{N}\;s_{i}}{\left(1-q\right)}^{N-\sum_{i=1}^{N}\;s_{i}}, \end{array}$$ (5)

where *q* is the probability of eliciting a spike. For such population,

$$\begin{array}{c} \frac{1}{N}\sigma \;(\log p_{N}\left(s\right))=\frac{1}{\sqrt{N}}\sqrt{q(1-q)}\log\frac{q}{1-q}, \end{array}$$ (6)

which vanishes for very large number of neurons, and so the system is not critical. More generally, if *p*_*N*_(**s**) can be factorized into a product of probability distributions over smaller subpopulations which are independent of each other and whose number is proportional to *N*, then log *p*_*N*_(**s**)/*N* turns into an empirical average whose standard deviation is expected to vanish in the large *N* limit, and the population is not critical. Reversing this argument, signatures of criticality can be interpreted as evidence that the population is globally coupled, i.e. that it cannot be decomposed into independent parts.

These preliminaries establish a direct link between criticality and semiparametric energy models of Eq (3). Nonlinearity in semiparametric energy models makes sure that the statistics of the energy *E*(**s**; ***α***), and, since *V* (*E*) is monotone, also the statistics of log *p*(**s**; ***α***, *V*) are modeled accurately (see Methods). Because the behavior of log probability is crucial for criticality, as argued above, semiparametric energy models can capture accurately and efficiently the relevant statistical structure of any system that exhibits signs of criticality and/or global coupling.

### Nonparametric estimation of the nonlinearity

To fully specify semiparametric energy models, we need a procedure for constructing the nonlinearity *V* (*E*). We cannot let this function be arbitrary because then the model could learn to assign nonzero probabilities only to the samples in the dataset, and hence it would overfit. To avoid such scenarios, we will restrict ourselves to functions which are increasing. We also require *V* (*E*) to be differentiable so that we can utilize its derivatives when fitting the model to data. The class of increasing differentiable functions is very large. It includes functions as diverse as the sigmoid, 1/(1 + exp(−*E*)), and the square root, $\sqrt{E}$ (for positive *E*), but we do not want to restrict ourselves to any such particular form—we want to estimate *V* (*E*) nonparametrically.

Nonparametric estimation of monotone differentiable functions is a nontrivial yet very useful task (for example, consider tracking the height of a child over time—the child is highly unlikely to shrink at any given time). We follow Ref [36] and restrict ourselves to the class of strictly monotone twice differentiable functions for which *V*′′/*V*′ is square-integrable. Any such function can be represented in terms of a square-integrable function *W* and two constants *γ*_1_ and *γ*_2_ as

$$\begin{array}{c} V\left(E\right)=\gamma_{1}+\gamma_{2}\;\int_{E_{0}}^{E}\;\exp(\;\int_{E_{0}}^{E^{\prime }}\;W\left(E^{\prime \prime }\right)\;dE^{\prime \prime })\;dE^{\prime }, \end{array}$$ (7)

where *E*_0_ is arbitrary and sets the constants to *γ*_1_ = *V* (*E*_0_), *γ*_2_ = *V*′(*E*_0_). The function is either everywhere increasing or everywhere decreasing (depending on the sign of *γ*_2_) because the exponential is always positive. Eq (7) is easier to understand by noting that *V* (*E*) is a solution to the differential equation *V*′′ = *WV*′. This means, for example, that on any interval on which *W* = 0, the equation reduces to *V*′′ = 0, and so *V* (*E*) is a linear function on this interval. If *V* (*E*) is increasing (*V*′ > 0), it also shows that the sign of *W* at a given point determines the sign of the second derivative of *V* at that point.

An advantage of writing the nonlinearity in the form of Eq (7) is that we can parametrize it by expanding *W* in an arbitrary basis without imposing any constraints on the coefficients of the basis vectors yet *V* (*E*) is still guaranteed to be monotone and smooth. In particular, we will use piecewise-constant functions for W. This allows us to use unconstrained optimization techniques for fitting our models to data.

## Results

We analyzed a simultaneous recording from 160 neurons in a salamander retina which was presented with 297 repetitions of a 19 second natural movie. The data was collected as part of a previous study [11], and is publicly available [37]. All models were trained using a variation of Persistent Contrastive Divergence [38] which performs an approximate gradient ascent on the log-likelihood of data. The nonparametric estimate of *V* only added 20 additional parameters to each model, and the gradient ascent learned these parameters simultaneously with the parameters of the energy function. Details regarding the parametrization of *V* and the algorithm for learning models from data can be found in Methods, and our code is available at https://github.com/jhumplik/generative-neural-models.

The population responses were binary vectors **s** ∈ {0, 1}^*N*^ representing which neurons elicited an action potential during a 20 ms time window. All responses were pooled across time and repeats; hence, we did not utilize the repeat structure in any way during model inference. For some analyses we examined the scaling of various quantities of interest with the population size. To this end, we used our data to construct 30 smaller datasets as follows. We randomly select 40 neurons from the total of 160 as the first dataset. Then we augment this dataset with 20 additional neurons to yield the second dataset, and we keep repeating this process until we have a dataset of 140 neurons. This whole process is repeated 5 times, resulting in 5 datasets for each of the 6 different population sizes. For each dataset, we set aside responses corresponding to randomly selected 60 (out of 297) repetitions of the movie, and use these as test data.

### Semiparametric independent model

We start by considering one of the simplest models of the form Eq (3), the *semiparametric independent model*:

$$\begin{array}{c} p\left(s;\alpha ,V\right)=\frac{e^{-V\;(-\sum_{i=1}^{N}\;\alpha_{i}s_{i})}}{Z(\alpha ,V)}. \end{array}$$ (8)

If *V* were a linear function, the model would reduce to an *independent model*, i.e. a population of independent neurons with diverse firing rates. In general, however, *V* introduces interactions between the neurons that may not have a straightforward low-order representation. When fitted to our data, the nonlinearity *V* turns out to be a concave function (see later sections on more complex models for a detailed discussion of the shape of the nonlinearity). Note that if *V* had a simple functional form such as a low order polynomial, then the model Eq (8) would be closely related to mean field models of ferromagnetism with heterogenous local magnetic field studied in physics.

**Fig 2:**
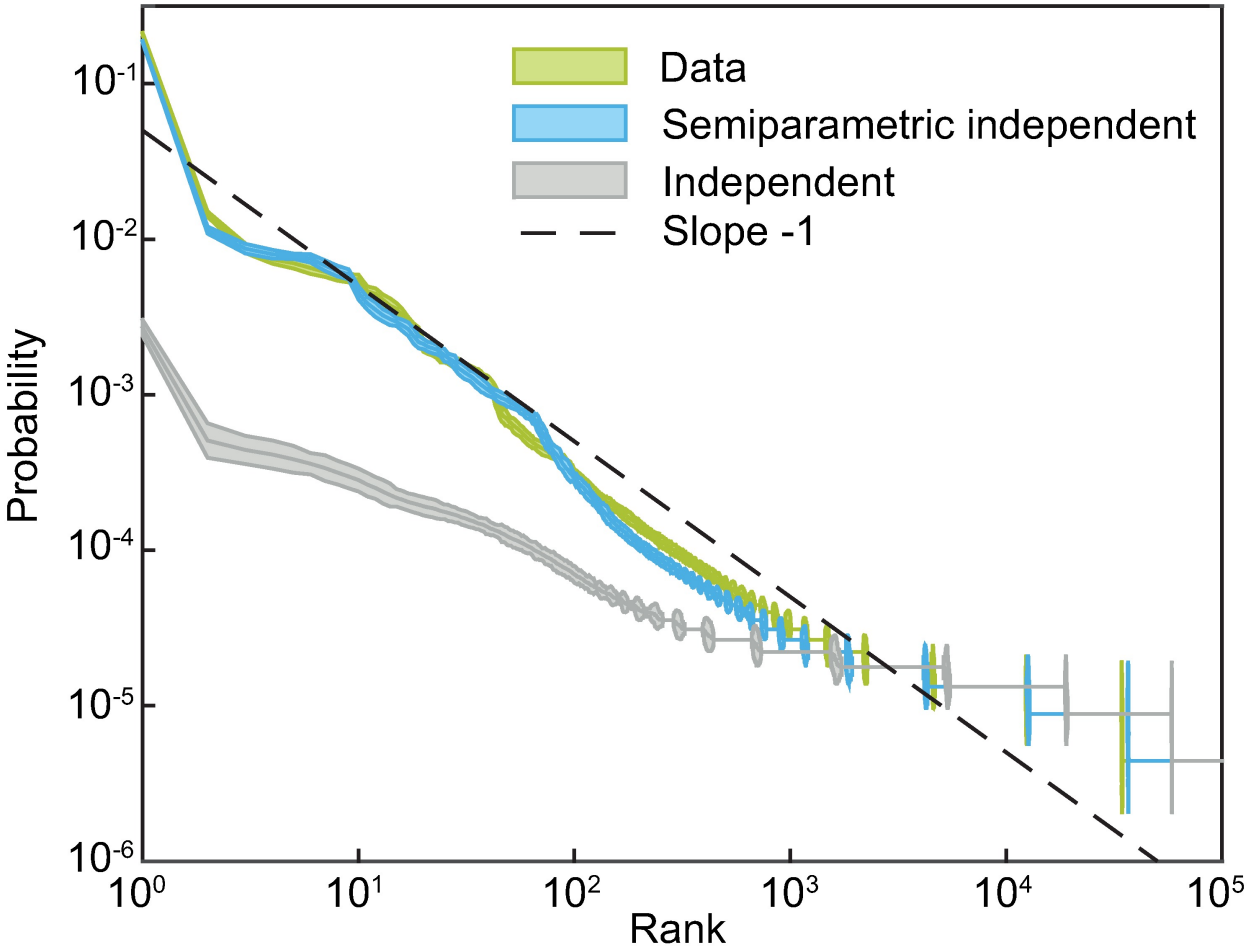
Semiparametric independent model reproduces the empirical Zipf plot. Each curve shows the probabilities of population activity patterns, *P*(**s**), sorted in decreasing order on a log-log plot. To construct the empirical Zipf plot, we directly sampled the frequencies of different patterns from data. To construct model predictions, we used the same procedure but replaced real data with artificial datasets of the same size, generated by drawing the samples from the corresponding model. Error bars are 3 SD (bootstrapped).

Our first goal is to use this simple model to verify our intuition that the nonlinearity helps to capture criticality. Many population patterns are observed several times during the course of the experiment, and so it is possible to estimate their probability simply by counting how often they occur in the data [19]. Given this empirical distribution, we construct a corresponding Zipf plot—a scatter plot of the frequency of a pattern vs its rank. For systems which are close to critical, this should yield a straight line with slope close to −1 on a log-log scale. We repeat the same procedure with samples generated from a semiparametric independent model as well as an independent model, which were both fitted to the responses of all 160 neurons. Fig 2 shows all three scatter plots. The independent model vastly deviates from the empirical Zipf plot; specifically, it greatly underestimates the probabilities of the most likely states. In contrast, the learned semiparametric independent model follows a similar trend to that observed in data. This does not mean that the semiparametric independent model itself is an excellent model for the detailed structure in the data, but it is one of the simplest possible extensions of the trivial independent model that qualitatively captures both global coupling and the signatures of criticality.

**Fig 3:**
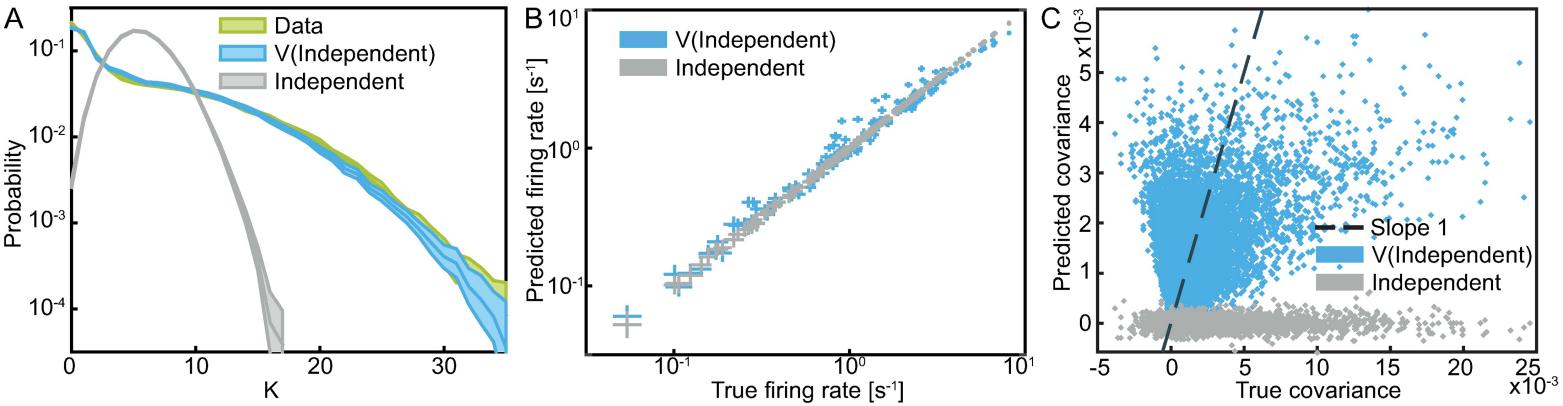
Comparison of the semiparametric independent and the independent model. **A)** Probability distributions of the total activity of the population, *K*(**s**) = ∑_*i*_ *s*_*i*_, estimated from data and from model samples. Error bars are 3 SD (bootstrapped), with the model-generated sample size equal to that of the data. **B)** Comparison of the firing rates estimated from the data and from the model samples. The firing rates predicted by the independent model should exactly match the true firing rates. Error bars are 3 SD (bootstrapped). **C)** Comparison of the predicted pairwise covariance matrix elements estimated from the model and from data, for the semiparametric independent and the independent models. The scatter of independent model covariance elements around 0 illustrates the magnitude of the sampling noise.

Since the semiparametric independent model is able to capture the criticality of the data distribution, we also expect it to accurately model other features of the data which are related to the globally coupled nature of the population. To verify this, Fig 3A compares the empirical probability distribution of the total activity of the population *K*(**s**) = ∑_*i*_ *s*_*i*_ to that predicted by the semiparametric independent model. The match is very accurate, especially when compared to the same distribution predicted by the independent model. This result goes hand in hand with the analysis in [39] which showed that interactions of all orders (in our case mediated by the nonlinearity) are necessary to model the wide-spread distribution of the total activity.

The independent model is a maximum entropy model which constrains the mean responses, 〈*s*_*i*_〉, of all neurons. In other words, neurons sampled from the model would have the same firing rates as those in the data (up to sampling noise). Even though the semiparametric independent model is strictly more general, it does not retain this property when the parameters ***α*** and the nonlinearity *V* are learned by maximizing the likelihood of data. Fig 3B demonstrates this point: although the predicted firing rates are approximately correct, there are slight deviations. On the other hand, the nonlinearity induces pairwise correlations between neurons which is something the independent model by construction cannot do. Fig 3C compares these predicted pairwise correlations to their data estimates. While there is some correlation between the predicted and observed covariances, the semiparametric independent model often underestimates the magnitude of the covariances and does not capture the fine details of their structure (e.g. the largest covariance predicted by the semiparametric independent model is about 5× smaller than the largest covariance observed in the data). This is because a combination of independent terms and a single nonlinearity does not have sufficient expressive power, motivating us to look for a richer model.

### Semiparametric pairwise model

One way to augment the power of the semiparametric independent model that permits a clear comparison to previous work is by means of the semiparametric pairwise model:

$$\begin{array}{c} p\left(s;J,V\right)=\frac{1}{Z(J,V)}\exp\;(-V\;(-\sum_{i,j=1}^{N}\;J_{ij}s_{i}s_{j})). \end{array}$$ (9)

**Fig 4:**
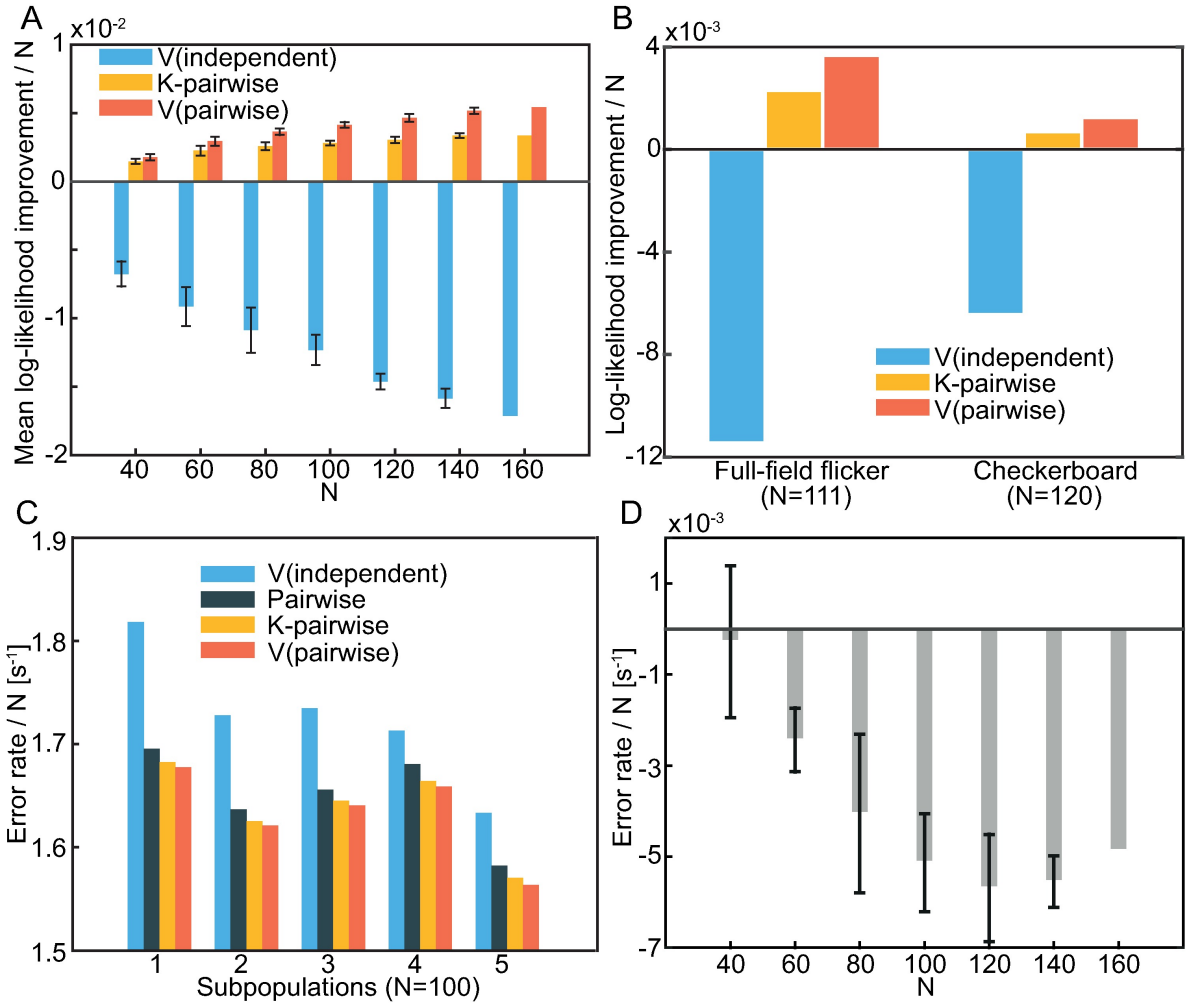
Semiparametric pairwise model outperforms other models. **A)** Out-of-sample log-likelihood improvement relative to the pairwise model per sample per neuron averaged over subnetworks. Error bars denote variation over subnetworks (1 SD, no errorbars for *N* = 160 since there is only one subpopulation of that size in the entire dataset). The error in likelihood estimation is much smaller than the displayed error bars. **B)** The same as in A) but for single populations from two different experiments–one in which the population is stimulated with a random checkerboard stimulus, and the other where the population responds to a full-field flickering. **C)** The test set error rate averaged over neurons for predicting the response of a neuron from the activities of other neurons in 5 different subpopulations of 100 neurons. **D)** Average (across neurons) error rate decrease achieved by using a semiparametric pairwise model instead of a K-pairwise model for subpopulations of various sizes. Error bars denote 1 SD variation over subnetworks.

We fit this model to the responses of the various subpopulations of the 160 neurons, and we compare the resulting goodness-of-fit to that of a pairwise (Eq (1)), K-pairwise (Eq (2)), and semiparametric independent model (Eq (8)). We measure goodness-of-fit as the improvement of the log-likelihood of data per neuron under the model relative to the pairwise model, as shown in Fig 4A. This measure reflects differences among models rather than differences among various subpopulations. The semiparametric pairwise model consistently outperforms the other models and this difference grows with the population size. To make sure that this improvement is not specific to this particular experiment, we also fitted the models to two additional recordings from the salamander retina which were also collected as part of the study [11]. One consists of 120 neurons responding to 69 repeats of a 30 second random checkerboard stimulus, and the other of 111 neurons responding to 98 repeats of a 10 second random full-field flicker stimulus. As shown in Fig 4B, the improvements of individual models on these datasets are consistent with the ones observed for the population stimulated with a natural movie.

The advantage of using likelihood as a goodness-of-fit measure is its universal applicability which, however, comes hand-in-hand with the difficulty of interpreting the quantitative likelihood differences between various models. An alternative comparison measure that has more direct relevance to neuroscience asks about how well the activity of a single chosen neuron can be predicted from the activities of other neurons in the population. Given any probabilistic model for the population response, we use Bayes rule to calculate the probability of the *i*th neuron spiking (*s*_*i*_ = 1) or being silent (*s*_*i*_ = 0) conditioned on the activity of the rest of the population (**s**_−*i*_) as

$$\begin{array}{c} p\left(s_{i}|s_{-i};\alpha \right)=\frac{p(s;\alpha )}{p\left(s_{i}=1,s_{-i};\alpha \right)+p\left(s_{i}=0,s_{-i};\alpha \right)}. \end{array}$$ (10)

We turn this probabilistic prediction into a nonrandom one by choosing whether the neuron is more likely to spike or be silent given the rest of the population, i.e.

$$\begin{array}{c} s_{i}\left(s_{-i};\alpha \right)=\underset{s_{i}\in \left\{0,1\right\}}{\mathrm{argmax}}p\left(s_{i}|s_{-i};\alpha \right). \end{array}$$ (11)

In Fig 4C and 4D we compare such predictive single neuron models constructed from semiparametric pairwise, K-pairwise, pairwise, and semiparametric independent models learned from the data for populations of various sizes. Specifically, we ask how often these models would make a mistake in predicting whether a chosen single neuron has fired or not. Every population response in our dataset corresponds to 20 ms of an experiment and so we can report this accuracy as number of errors per unit of time. Predictions based on the semiparametric pairwise model are consistently the most accurate.

**Fig 5:**
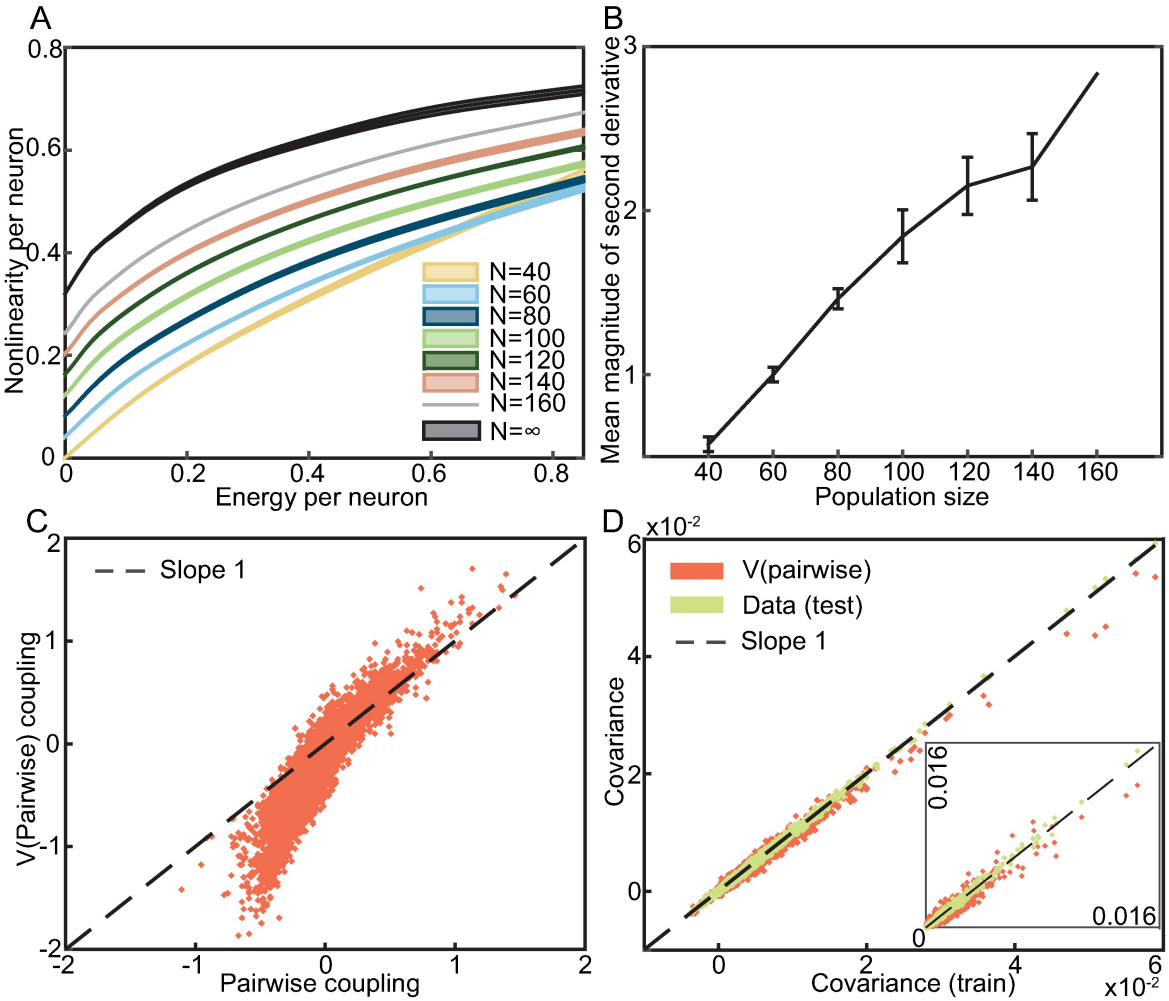
Properties of the semiparametric pairwise model. **A)** Plot of *V* (*E*) vs *E*, i.e. the inferred nonlinearities of the semiparametric pairwise model. Curves are normalized by network size *N* and shifted along the y-axis for readability. Error bars (1 SD) denote variation over different subnetworks. The black curve is an extrapolation of the other curves to a large population size. **B)** The population size dependence of the average absolute value of the nonlinearity’s second derivative. Error bars (1 SD) denote variation over different subnetworks. **C)** Scatter plot of the couplings from a semiparametric pairwise model vs those from a pairwise model fitted to the whole population of 160 neurons. **D)** Comparison of the covariances predicted by the semiparametric pairwise model vs. those estimated from the training data. As an approximate guide for the sampling noise, covariances estimated from test data are also compared to covariances estimated from training data. Inset shows the same plot but with 10000 randomly sampled third moments **E**[*s*_*i*_*s*_*j*_*s*_*k*_] such that *i* ≠ *j* ≠ *k* instead of the covariances.

Fig 5A shows the nonlinearities of the semiparametric pairwise models that we learned from data. In order to compare the nonlinearities inferred from populations of various sizes, we normalize the domain of the nonlinearity as well as its range by the number of neurons. Even though the nonlinearities could have turned out to have e.g. a sigmoidal shape, the general trend is that they are concave functions whose curvature—and thus departure from the linear *V* that signifies no global coupling—grows with the population size. The shape of these nonlinearities is reproducible over different subnetworks of the same size with very little variability. To further visualize the increasing curvature, we extrapolated what these nonlinearities might look like if the size of the population was very large (the black curve in Fig 5A). This extrapolation was done by subtracting an offset from each curve so that *V*(0) = 0, and then fitting a straight line to a plot of 1/*N* vs. the value of *V* at points uniformly spaced in the function’s domain. The plots of 1/*N* vs. *V* are only linear for *N* ≥ 80, and so we only used these points for the extrapolation which is read out as the value of the fit when 1/*N* = 0. To quantify the increasing curvature, Fig 5B shows the average absolute value of the second derivative of *V* across the function’s domain.

The coupling matrix ***J*** of both the pairwise and the semiparametric pairwise models describes effective interactions between neurons, and so it is interesting to ask how the couplings predicted by these two models are related. While Fig 5C shows a strong dependency between the couplings in a network of *N* = 160 neurons, the dependency is not deterministic and, moreover, negative couplings tend to be amplified in the semiparametric pairwise model as compared to the pairwise model. Similarly to the semiparametric independent model, there is no guarantee that the semiparametric pairwise model will reproduce observed pairwise correlations among neurons exactly, even though pairwise model has this guarantee by virtue of being a maximum entropy model. Fig 5D shows that despite the lack of such a guarantee, semiparametric pairwise model predicts a large majority of the correlations accurately, with the possible exceptions of several very strongly correlated pairs. This is simply because the semiparametric paiwise model is very accurate–the inset of Fig 5D shows that it can also reproduce third moments of the responses. A K-pairwise model also has this capability but, as shown in Ref [11], a pairwise model systematically mispredicts higher than second moments.

### Shape of the nonlinearity in critical models

Suppose we use the semiparametric pairwise model to analyze a very large population which is not globally coupled and can be divided into independent subpopulations. The only way the model in Eq (9) can be factorized into a product of probability distributions over the subpopulations is if the function *V* is linear. Therefore, the prior knowledge that the population is not globally coupled immediately implies the shape of the nonlinearity. Similarly, a prior knowledge that the population is critical also carries a lot of information about the shape of the nonlinearity.

We show in Methods that if the parameters ***α*** are known, then the optimal nonlinearity in Eq (3) can be explicitly written as

$$\begin{array}{c} V\left(E\right)=\log\bar{\rho }\left(E;\alpha \right)-\log\hat{\bar{p}}\left(E;\alpha \right), \end{array}$$ (12)

where $\bar{\rho }\left(E;\alpha \right)$ is the *density of states* which counts the number of patterns **s** whose energy is within some narrow range [*E*, *E* + Δ]. The density of states is a central quantity in statistical physics that can be estimated also for neural activity patterns either directly from data or from inferred models [19]. Similarly, $\hat{\bar{p}}\left(E;\alpha \right)$ is the empirical probability density of the energy *E*(**s**; ***α***) smoothed over the same scale Δ. Eq (12) follows from the relation $\hat{\bar{p}}\left(E;\alpha \right)\propto \bar{\rho }\left(E;\alpha \right)\exp(-V\left(E)\right)$, i.e. the probability of some energy level is just the number of states with this energy times the probability of each of these states (see Methods).

We would like to establish a prior expectation on what the large *N* limit of the nonlinearites in Fig 5A is. Adapting the same normalization as in the figure, we denote *ϵ*(**s**; ***α***) = *E*(**s**; ***α***)/*N*. Changing variables and rewriting Eq (12) in terms of the empirical probability density of the normalized energy $\hat{\bar{p_{\epsilon }}}\left(\epsilon \right)=N\hat{\bar{p}}\left(\epsilon N;\alpha \right)$ yields

$$\begin{array}{c} V\;\left(\epsilon N\right)=\log\bar{\rho }\left(\epsilon N;\alpha \right)-\log\hat{\bar{p_{\epsilon }}}\left(\epsilon \right)+\log N. \end{array}$$ (13)

For a system where *s*_*i*_ can take on two states, the total number of possible activity patterns is 2^*N*^, and so we expect the log of the density of states to be proportional to *N*. If the system is critical, then by virtue of Eq (4) *σ*(log *p*_*N*_(**s**)) is proportional to N, and similarly we also expect *σ*(*E*(**s**; ***α***)) ∝ *N*. This means that *σ*(*ϵ*(**s**; ***α***)) = *σ*(*E*(**s**; ***α***))/*N* converges to some finite, nonzero number, and therefore $\log\hat{\bar{p_{\epsilon }}}\left(\epsilon \right)$ also stays finite no matter how large the population is. Taken together, for large critical populations, the first term on the right hand side of Eq (13) is the only one which scales linearly with the population size, and hence it dominates the other terms:

$$\begin{array}{c} V\left(E\right)\approx \log\bar{\rho }\left(E;\alpha \right). \end{array}$$ (14)

One of our important results is thus that for large critical populations, the nonlinearity should converge to the density of states of the inferred energy model. In other words, for critical systems as defined in Eq (4), there is a precise matching relation between the nonlinearity *V* (*E*) and the energy function *E*(**s**; ***α***); in theory this is exact as *N* → ∞, but may hold approximately already at finite *N*.

**Fig 6:**
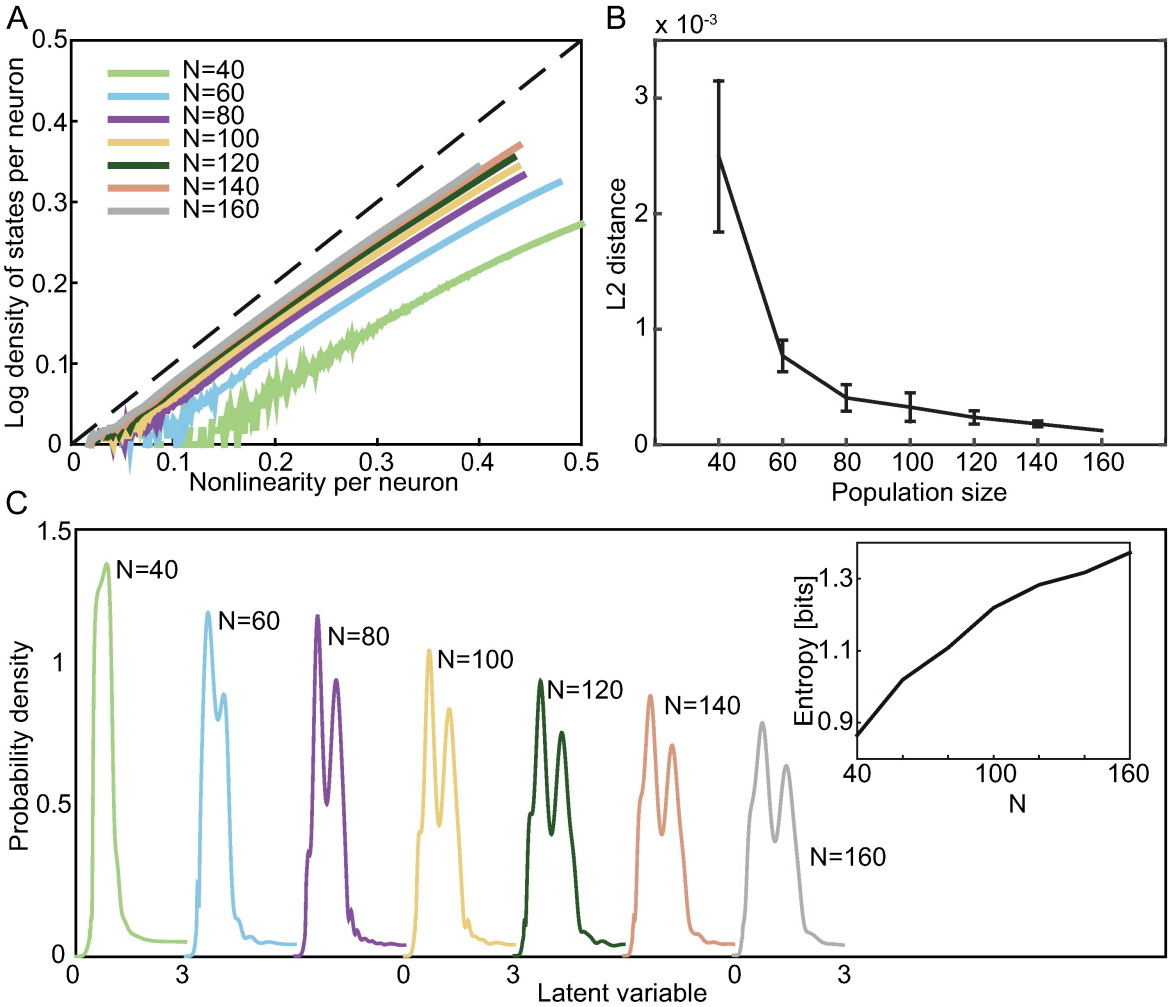
Properties of the inferred nonlinearity for neural networks of increasing size. **A)** Comparison between the inferred nonlinearity in the range of energies observed in the dataset and the log of the density of states at the same energies, showing the increasing match between the two quantities as the population size, *N*, increases. Both axes are normalized by the population size so that all curves have a similar scale. Nonlinearity can be shifted by an arbitrary constant without changing the model; to remove this redundancy, we set *V* (0) = 0 for all nonlinearities. **B)** The population size dependence of the average squared distance between the density of states and the inferred nonlinearity. Since the nonlinearity can be shifted by an arbitrary constant, we chose this offset so as to minimize the average squared distance. Error bars (1 SD) denote variation over different subnetworks. **C)** Inferred nonlinearities map to latent variables whose probability distributions can be computed and plotted for one sequence of subnetworks increasing in size (colors). As the network size increases, the dynamic range of the latent variable distribution does as well, which is quantified by the entropy of the distributions (inset).

To verify that this is the case for our neural population that has previously been reported to be critical, we compare in Fig 6A the nonlinearity inferred with the semiparametric pairwise model (Fig 5A) to the density of states estimated using a Wang and Landau Monte Carlo algorithm [40] for a sequence of subpopulations of increasing size. As the population size increases, the nonlinearity indeed approaches the regime in which our prediction in Eq (14) holds. This convergence is further quantified in Fig 6B which shows the average squared distance between the density of states and the nonlinearity. The average is taken over the range of observed energies. The nonlinearities are only specified up to an additive constant which we chose so as to minimize the squared distance between the density of states and the nonlinearity.

### Mapping the nonlinearity to a latent variable

The link between global coupling and criticality is related to recent theoretical suggestions [28, 29], where global coupling between the neurons in the population emerges as a result of shared latent (fluctuating) variables that simultaneously act on extensive subsets of neurons. In particular, Ref [28] theoretically analyzed models with a multivariate continuous latent variable **h** distributed according to some probability density *q*(**h**), whose influence on the population is described by the conditional probability distribution

$$\begin{array}{c} p_{N}\left(s|h\right)=\frac{e^{-\sum_{j}\;h_{j}O_{j}^{\left(N\right)}\left(s\right)}}{Z_{N}\left(h\right)}, \end{array}$$ (15)

where *Z*_*N*_(**h**) is a normalization constant, and $O_{j}^{\left(N\right)}\left(s\right)$ are global quantities which sum over the whole population. The authors showed that under mild conditions on the probability density *q*(**h**) of **h**, and the scaling of $O_{j}^{\left(N\right)}\left(s\right)$ with *N*, the sequence of models

$$\begin{array}{c} p_{N}\left(s\right)=\int \;q\left(h\right)p_{N}\left(s|h\right)\;dh \end{array}$$ (16)

is critical in the sense of Eq (4).

If the latent variable is one-dimensional, i.e. **h** = *h*, then the models in Eq (16) have exactly the form of models in Eq (3) with *E*(**s**; ***α***) = *O*(**s**), i.e. given a probability density *q*(*h*) of the latent variable, we can always find a nonlinearity *V* (*E*) such that

$$\begin{array}{c} \frac{1}{Z(\alpha )}e^{-V(E(s;\alpha ))}=\int_{0}^{\infty }\;q\left(h\right)\frac{e^{-hE(s;\alpha )}}{Z(h;\alpha )}\;dh. \end{array}$$ (17)

The reverse problem of finding a latent variable for a given function *V* (*E*) such that this equation is satisfied does not always have a solution. The condition for this mapping to exist is that the function exp(−*V* (*E*)) is totally monotone [41], which, among other things, requires that it is convex. While our models allow for more general nonlinearites, we showed in Fig 5A that the inferred functions *V* (*E*) are concave and so we expect this mapping to be at least approximately possible (see below).

The mapping in Eq (17) is based on a Laplace transformation, a technique commonly used for example in the study of differential equations. Laplace transformations are also often used in statistical physics where they relate the partition function of a system to its density of states. While the mathematics of Laplace transformations yields conditions on the function *V* (*E*) so that it is possible to map it to a latent variable (i.e., exp(−*V* (*E*)) must be totally monotone), analytically constructing this mapping is possible only in very special cases. We can gain a limited amount of intuition for this mapping by considering the case when the latent variable *h* is a narrow gaussian with mean *h*_0_ and variance *σ*^2^. For small *σ*^2^, one can show that

$$\begin{array}{c} V\left(E\right)\approx h_{0}E-\sigma^{2}{\left(E-E_{0}\right)}^{2}, \end{array}$$ (18)

where *E*_0_ is the average energy if *σ*^2^ = 0, and the approximation holds only in a small neighborhood of *E*_0_ (|*E* − *E*_0_| ≪ *σ*). This approximation shows that the curvature of *V* (*E*) is proportional to the size of the fluctuations of the latent variable which, in turn, is expected to correlate with the amount of global coupling among neurons.

This relationship to global coupling can be understood from the right hand side of Eq (17). When the energy function is, for example, a weighted sum of individual neurons as in the semiparametric independent model of Eq (8), then we can think of Eq (17) as a latent variable *h* (perhaps reflecting the stimulus) coupled to every neuron, and hence inducing a coupling between the whole population. A non-neuroscience example is that of a scene with **s** representing the luminance in each pixel, and the latent *h* representing the lighting conditions which influence all the pixels simultaneously.

We used the right hand side of Eq (17) (see Methods) to infer the shapes of the probability densities of the latent variables which correspond to the nonlinearities in the semiparametric pairwise models learned from data. These probability densities are shown in Fig 6C. A notable difference to the formulation in Eq (16) is that the inferred latent variables scale with the population size; in particular, the inset to Fig 6C shows that the entropy of the inferred latent variable increases with the population size. Entropy is a more appropriate measure of the “broadness” of a probability density than standard deviation when the density is multimodal. Taken together with the results in Fig 4A, this suggests that global coupling is especially important for larger populations. However, it is also possible that the latents are becoming broader because the model is trying to compensate for limited capacity, and that the entropy of the latent would saturate if we had a more expressive energy function. Larger datasets and/or further improvements in probabilistic models are necessary to make more detailed conclusions.

**Fig 7:**
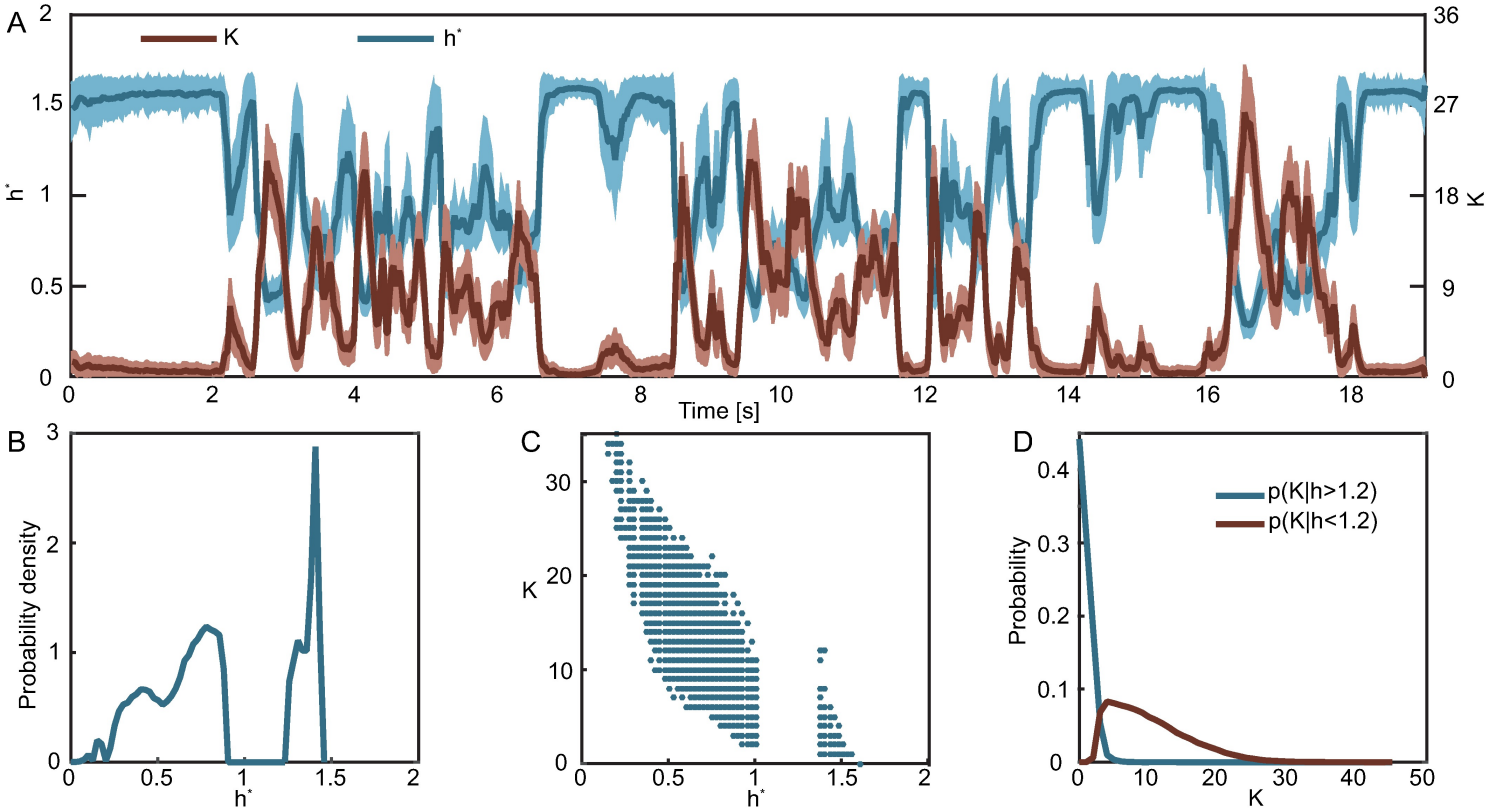
The most likely value of the latent variable naturally defines two global population states. **A)** For every repeat of the stimulus and for every time bin we estimate the most likely value of the latent (h*) given the population response at that time, as well as the total number of spiking neurons in that response (*K*). The plot shows the trajectories of h* and *K* averaged across repeats. Error bars correspond to 1 SD. **B)** Probability density of h*, i.e. the most likely value of the latent given the population response. **C)** A scatter plot of the total network activity vs. the most likely value of the latent. h* naturally divides the population responses into two clusters. **D)** Probability distribution of the total network activity given this global population state. While the most likely value of *K* for low h* is zero, the distribution has a tail that extends to *K* ≈ 5.

Interestingly, the probability densities of the latent variables consist of two modes at approximately *h* = 0.7 and *h* = 1.3. We hypothesize that these modes reflect a discrete-like nature of the population dynamics which consist of bursts of activity interspaced with periods of approximate silence. These bursts are demonstrated in Fig 7A where we show the time dependence of the total network activity. Unfortunately, closer inspection reveals that the total network activity cannot be used in a straightforward manner to classify the population as active or inactive. The reason is that neurons are noisy and if we defined a population as inactive when the total network activity is 0, then such definition is not robust to noise. In fact, the probability distribution of the total network activity (Fig 3A) is such that there is no obvious choice of a threshold, and so quantifying the discreteness of the population dynamics based on the total network activity would be arbitrary.

To circumvent these problems and enable a robust classification of the population state as active or inactive, we can use the most likely value of the latent variable given a population response, i.e.

$$\begin{array}{c} h^{*}\left(s\right)=\underset{h}{\mathrm{argmax}}p\left(h|s\right)=\underset{h}{\mathrm{argmax}}p\left(s|h\right)q\left(h\right)=\underset{h}{\mathrm{argmax}}q\left(h\right)\frac{e^{-hE(s;\alpha )}}{Z(h;\alpha )}. \end{array}$$ (19)

Fig 7A shows the time dependence of h*, and Fig 7B its probability density (estimated by collecting h*(**s**) over all repeats and times). The probability density of h* has two modes separated by an inaccessible region, so one can easily classify a population response **s** as active or inactive based on which mode h*(**s**) belongs to. Fig 7C and 7D show that a population pattern with, for example, 5 active neurons can have very different values for h*(**s**), demonstrating that any measure based on the total network activity would easily confuse which state the population is in.

## Discussion

Criticality is a theoretical concept which depends crucially on how the probability distribution over population activity patterns scales with the population size. Constructing this scaling directly from data is complicated, and necessarily involves extrapolating to large population sizes [10, 30]. As a consequence, answering the question whether a population is critical or “how close to critical” it is, is difficult. Here we took a different approach—we used the theoretical notion of criticality to guide our intuition about what models are useful for analyzing populations that exhibit signs of criticality such as an approximate Zipf law. From the standpoint of fitting statistical models, it is irrelevant whether or not the studied population is really critical given some operational realization of the large population size limit because our models can be used either way, and their accuracy can be evaluated using standard model selection techniques. In particular, our approach is agnostic to the origins of the signatures of criticality which have been hotly debated [25–30, 42]. Our reasoning is thus very pragmatic: we on purpose avoided the controversial (albeit interesting) issues of whether the observed critical behavior in real data is “trivial” or not and what may be its mechanistic explanation, and focused rather on making use of the observation itself to design better probabilistic models for neural code.

This pragmatic approach is driven by the rapid development of experimental techniques for recording the activity of large neural populations, which is posing a challenge for data analysis. The number of neurons that we can measure simultaneously is growing much faster than the time period over which we can record from these neurons. Therefore, we might soon be in a regime where the number of available samples is comparable to the population size. To make meaningful conclusions from such datasets, our models will need to take maximal advantage of the prior knowledge about the dependency structure among neurons. The prior knowledge that the distribution of activity could be close to critical and that the population could be globally coupled are two macroscopic features of the neural code that future models should be able to reproduce without extreme tuning of many parameters. Our semiparametric energy models directly utilize this prior knowledge, and because the complexity of the nonlinearity is held fixed for all population sizes, it can be easily used in models with arbitrary number of neurons.

While today’s neuroscience provides us with sufficient data to build accurate models of neural populations, it is also important that these models generate new hypotheses and shape the direction of future research. For example, our goal was not to trace the origins of the observed Zipf law, but we nevertheless believe that the pursuit of these origins can only happen in a data-driven context to which our models will further contribute. There are many toy models that reproduce Zipf law, several of which have been proposed in the neuroscience context to additionally account for related signatures of criticality, e.g., the behavior of the heat capacity. Some of these models invoked the particular structure of the observed pairwise correlations, ascribed specific importance to fluctuating (latent) variables (see Discussion in [19]) which could (or not) be directly related to the stimulus itself, or suggested that the processes of model construction, inference, or scaling to large *N* generate spurious signatures of criticality. The issue is thus not the lack of possible explanations. Rather, it is that these explanations account qualitatively for only one selected aspect of the actual data, while not truly testing whether the proposed explanation is quantitatively consistent with *all* of the reported phenomena and measured statistics. Here, we took seriously the idea that the signatures of criticality could be due to a global coupling to a hidden (latent) fluctuating variable, as proposed and discussed in the context of a blowfly motion-sensitive neuron in Ref [28], and we have shown that the proposed mechanism is viable in a model that precisely accounts for a real and well-studied dataset [11].

It is important to stress that the identified latent variable is only an effective description of the data, and so, without further experiments, we cannot interpret it in terms of some biophysical mechanisms, nor can we claim, for example, that the population is critical because of this latent variable. However, knowing that this latent variable is a useful statistic describing the population should be a motivation for designing future experiments so that we can correlate it with more detailed mechanisms on the level of neural circuits, and possibly gain insight into its bimodal structure. It also suggests that we should analyze populations responding to various stimuli so that we can understand the latent variable’s stimulus dependence. The scaling of the latent variable shown in Fig 6C also suggests that we should reexamine whether we could find even better description of the data with more than one latent variables. This could be done by studying models with multiple or with multidimensional nonlinearities. Generally, these models have the form log *p*(**s**) ∝ *V* (*E*_1_(**s**), *E*_2_(**s**), …), and a particularly interesting special case is when each “energy” function *E*_*i*_ is a simple linear projection of the responses as in the semiparametric independent model. These models offer an avenue for both improving the accuracy and reducing the number of parameters. In light of the theoretical analysis in Ref [28], each dimension of the nonlinearity could possibly be interpreted as a separate latent variable. While we are not aware of general conditions which would guarantee that a multidimensional nonlinearity can be mapped to a multidimensional latent variable, intuition suggests that as the dimension of the nonlinearity increases, the space of nonlinearities which allow for this inversion becomes smaller. This means that if we fit a model with a general multidimensional nonlinearity to data, and we find that this nonlinearity can be mapped to a multidimensional latent variable, then it is an evidence that these latent variables can be correlated with actual physical mechanisms which can be sought for in future experiments.

There exist alternative ways of modeling global coupling (and thus likely capturing signatures of criticality) in neural populations. Hidden-Markov-Model-type (HMM) models have been considered for the retinal data [43], where the discrete hidden states correspond to collective modes of activity that, due to noise in neural spiking, map probabilistically into observed activity patterns of spiking and silence. In contrast, our model can be interpreted as having a single (but continuous) hidden variable—although we empirically find that the distribution of this latent variable is actually bimodal, highlighting the basic distinction between the “silent” or “inactive” state of the retina, and the “active” state [44]. The HMM models were introduced to capture more flexibly collective modes of activity first observed in pairwise and K-pairwise models [10, 11]. Unlike the semiparametric pairwise model, they take into account the observed temporal dynamics, and they are also parametrically richer. Furthermore, their learned hidden states show interesting correspondence to the displayed stimuli even though the model is a priori agnostic about the stimulus. On the other hand, the HMM models admit no clear link to and interpretations of the signatures of criticality, which was our motivation in this paper. Related to the HMMs, [45, 46] discuss another classes of accurate models which capture the temporal dynamics of the population.

Unlike HMMs and related models, this paper is concerned with modeling the stationary distribution rather than the precise time-dependence of the population. While this discards a lot of information, and hence the resulting models are possibly less accurate, there are advantages to focusing on stationary models. On the technical side, temporal models require more parameters and associated decisions about how to represent the stimulus and its interactions with the population, and so they are harder to scale to datasets with large numbers of neurons. More importantly, however, it was precisely by disregarding the temporal information that the ubiquity of criticality and the role of weak pairwise correlations [8] in neural populations were discovered. It is thus possible that discarding the temporal information allows us to make more general observations about neural codes. This is an important hypothesis. For example, the models we consider in this work, as well as most of published models, are accurate only when applied to data collected in a very narrow experimental context, and it is unclear if/how much would these models generalize to novel stimuli/experimental conditions, nor is it obvious how to design experiments so that we can infer models which generalize as much as possible. While it remains to be tested, it is an intriguing hypothesis that stationary models have more potential for generalization across experiments.

In the domain of stationary models, Restricted Boltzmann Machines (RBMs) and their derivatives [34] are also classes of energy-based models for population activity that could capture global coupling by latent variables. RBMs are universal learners that, given sufficient data, can reproduce any distribution—including a critical one; like HMM models, however, making a generic link between their parameters and criticality appears difficult. We note that the RBM structure is not incompatible with the structure of semiparametric energy-based models: one could consider a “semiparametric RBM model,” where *E* in Eq 3 is defined by a RBM, whose parameters are learned jointly with the nonlinearity, *V* (*E*).

A different class of models that has been demonstrated to capture criticality consists of various derivatives of the dichotomized Gaussian model [26, 39, 47]. A comparison between the dichotomized Gaussian, pairwise, and K-pairwise models on the same dataset as we consider in this work was done in [11]. They showed that while the dichotomized Gaussian is comparable to the pairwise model, the K-pairwise model, and hence also the semiparametric pairwise model, are more accurate. The analysis in [48, 49] shows that the distribution of the total network activity (as in Fig 3) can often be fitted using a generalization of the dichotomized Gaussian model in which the inputs are q-Gaussians, but they assume that all neurons are the same and do not aim to model more detailed statistics of the neural responses. More recently [50] discusses how to extend the dichotomized q-Gaussian model to heterogeneous populations. However, they only show how to use this model to match the observed means and pairwise correlations while keeping the *q* parameter fixed, and they do not discuss how to perform maximum likelihood inference on all parameters simultaneously. Since these studies on the dichotomized q-Gaussian model showed that the *q* parameter is relevant for statistics related to global coupling, it would be an interesting research direction to develop a procedure for maximum likelihood inference of this model, and compare it to the semiparametric pairwise model.

The observations of criticality in real data are not specific to neuroscience. Datasets in many other fields such as luminance in natural images [31], or amino acid sequences of proteins [33] have been shown to exhibit Zipf law. In particular, models of the form Eqs (3) and (17) have been used to model the statistics of small image patches under the name elliptically symmetric distributions and Gaussian scale mixtures [51, 52] although the motivation for using these models had nothing to do with criticality. These models are much easier to analyze than the models we consider in this paper because the variables *s*_*i*_ are continuous rather than discrete. Our discussion regarding Eq (14) and the prior expectations about the shape of the nonlinearity is valid even in the continuous case. In particular, elliptically symmetric distributions are essentially the same as our semiparametric pairwise models, Eq (9), only with continuous variables. Because *s*_*i*_ are continuous, we can analytically evaluate the density of states,

$$\begin{array}{c} \rho \left(E;J\right)\propto E^{\frac{N}{2}-1}, \end{array}$$ (20)

and so the optimal nonlinearity for an elliptically symmetric distribution fitted to a large system which exhibits criticality (e.g. image patches) is expected to be *V* (*E*) = (*N*/2 − 1) log *E* + const.

Another connection between our models and a substantial body of theoretical work is in the context of nonextensive statistical mechanics. Physicists have considered models of the form Eqs (3) and (17) as models of systems whose entropy grows sublinearly with the system size [53]. It is difficult to make these connections explicit because nonextensive statistical mechanics has been studied mostly through toy models rather than data-driven generative models that we examine here; furthermore, in the toy models the latent variables are usually assumed to converge to a delta function as the population size grows which is in stark contrast with our findings in Fig 6. Nevertheless, deepening the connection between models inferred from data, the maximum entropy formalism itself (e.g., considering the possibility that our semiparametric energy models of Eq (3) can be derived from the maximization of a generalized version of the standard entropy), and nonextensive statistical mechanics is an interesting topic for further research.

## Methods

### Relation of the nonlinearity to the probability density of the energy

Let *ρ*(*E*′; ***α***) = ∑_*s*_ *δ*_*E*′, *E*(**s**; ***α***)_ count the number of states which map to the same energy *E*′. The probability distribution of *E*(**s**; ***α***) when **s** is distributed according to Eq (3) is

$$\begin{array}{c} p\left(E^{\prime };\alpha ,V\right)=\sum_{s}\;p\left(s;\alpha ,V\right)\delta_{E^{\prime },E\left(s;\alpha \right)}=\frac{\rho \left(E^{\prime };\alpha \right)e^{-V(E^{\prime })}}{Z(\alpha ,V)}. \end{array}$$ (21)

Given data $D={\left\{s^{\left(i\right)}\right\}}_{i=1}^{M}$, let $\hat{p}\left(E^{\prime };\alpha \right)=\frac{1}{M}\;\sum_{i=1}^{M}\;\delta_{E^{\prime },E\left(s^{\left(i\right)};\;\alpha \right)}$ be the data distribution of the energy, and let Ω_*α*_ be the image of *E*(**s**; ***α***). The average log-likelihood of the data can be rewritten as

$$\begin{array}{c} \begin{array}{cc} L(\alpha ,V) & =-\log Z\left(\alpha ,V\right)-\frac{1}{M}\;\sum_{i=1}^{M}\;V\left(E\left(s^{\left(i\right)};\alpha \right)\right)\\ & =-\log Z\left(\alpha ,V\right)-\sum_{E^{\prime }\in \Omega_{\alpha }}\;\hat{p}\left(E^{\prime };\alpha \right)V\left(E^{\prime }\right)\\ & =-\sum_{E^{\prime }\in \Omega_{\alpha }}\;\hat{p}\left(E^{\prime };\alpha \right)\log\rho \left(E^{\prime };\alpha \right)+\sum_{E^{\prime }\in \Omega_{\alpha }}\;\hat{p}\left(E^{\prime };\alpha \right)\log p\left(E^{\prime };\alpha ,V\right), \end{array} \end{array}$$ (22)

where the third line follows by substituting the logarithm of Eq (21).

Eq (22) has a simple interpretation. The last term, which is the only one depending on *V*, is the average log-likelihood of the samples ${\left\{E\left(s^{\left(i\right)};\alpha \right)\right\}}_{i=1}^{M}$ under the model *p*(*E*; ***α***, *V*), and so, for any ***α***, the purpose of the nonlinearity is to reproduce the data probability distribution of the energy.

Our restriction that *V* is a twice differentiable increasing function can be seen as a way of regularizing learning. The last term in Eq (22) is the negative cross entropy between $\hat{p}\left(E;\alpha \right)$ and *p*(*E*; ***α***, *V*) and it is well known that this term is maximal if $\hat{p}\left(E;\alpha \right)=p\left(E;\alpha ,V\right)$. According to Eq (21), if *V* was arbitrary, then, for any ***α***, we can satisfy this equality with any (possibly infinite) function *V* such that

$$\begin{array}{c} V\left(E\right)=\log\rho \left(E;\alpha \right)-\log\hat{p}\left(E;\alpha \right)+\text{const.}\;\text{for}\;\text{all}\;E\in \Omega_{\alpha }. \end{array}$$ (23)

If the energy function assigns distinct energies to distinct states, then the choice in Eq (23) leads to a model which exactly reproduces the empirical distribution of data, and hence overfits.

An alternative way of regularizing would be to assume that *V* is a piecewise constant function. In that case, the analog of Eq (23) is

$$\begin{array}{c} V\left(E\right)=\log\bar{\rho }\left(E;\alpha \right)-\log\hat{\bar{p}}\left(E;\alpha \right)+\text{const.}, \end{array}$$ (24)

where, for every bin on which *V* is constant, the density of states $\bar{\rho }\left(E;\alpha \right)$ counts the number of states whose energy maps to this bin divided by the bin width. Similarly, the empirical energy density $\hat{\bar{p}}\left(E;\alpha \right)$ counts the number of samples whose energy maps to this bin divided by the bin width.

### Learning the models

All models were trained using a variation of Persistent Contrastive Divergence [38] which performs an approximate gradient ascent on the log-likelihood for any model of the form *p*(**s**; ***α***) = exp(−*F*(**s**; ***α***))/*Z*(***α***), where *F*(**s**; ***α***) is a computationally tractable function differentiable in the parameters ***α***, and *Z*(***α***) is a normalization constant. Given an initial guess of the parameters ***α***_0_, and a list of *M*_*s*_ samples drawn from *p*(**s**; ***α***_0_), the algorithm can be summarized as

for *t* ≔ 1 to *L*

  ***α***_*t*_ = ***α***_*t*−1_ + *η*(**E**[∇_***α***_ *F*(**s**; ***α***_*t*−1_)]_samples_*t*−1__ − **E**[∇_***α***_ *F*(**s**; ***α***_*t*−1_)]_data_)

  samples_*t*_ = GIBBS^*n*^ (samples_*t*−1_, **α**_*t*_)

where *L* is the number of iterations, *η* is the learning rate, **E**[⋅]_list_ denotes an average over the list of states, and GIBBS^*n*^ represents *n* applications of the Gibbs sampling transition operator.

Pairwise and K-pairwise models were trained using *η* = 1, *n* = 2*N*, and with initial parameters drawn from a normal distribution with 0 mean and 0.1 standard deviation. We iterated the algorithm two times, first with *L* = 10000, *M*_*s*_ = 3 × 10^4^, then with *L* = 10000, *M*_*s*_ = 3 × 10^5^. Semiparametric independent and pairwise models were trained using *η* = 5 × 10^−5^ for the parameters of the function *V* (see below), and *η* = 1 for all other parameters. We initialized the model with parameters corresponding to the learned independent (pairwise) models, and trained for *L* = 10000 iterations with *M*_*s*_ = 3 × 10^4^ samples.

The function V is parametrized through a function W (see Eq (7)). We use piecewise constant functions to parametrize *W*. Let [*E*_0_, *E*_1_] be an interval containing the range of energies *E*(**s**; ***α***) which we expect to encounter during learning. We divide the interval [*E*_0_, *E*_1_] into *Q* non-overlapping bins of the same width with indicator functions *I*_*i*_, i.e. *I*_*i*_(*E*) = 1 if *E* is in the *i*th bin, otherwise *I*_*i*_(*E*) = 0, and we set $W\left(E\right)\equiv W\left(E;\beta \right)=\sum_{i=1}^{Q}\;\beta_{i}I_{i}\left(E\right)$. We used *Q* = 20 bins in all experiments. This was a conservative choice: increasing *Q* did not result in a higher training or validation likelihood.

The integrals in Eq (7) can be carried out analytically for this choice of *W* yielding an exact expression for *V* as a function of ***γ*** and ***β***. For *E* < *E*_0_, we have *V* (*E*; *γ*, ***β***) = *γ*_1_ + *γ*_2_(*E* − *E*_0_). For *E* > *E*_0_ we have *V* (*E*; *γ*, ***β***) = *γ*_1_ + *γ*_2_ *f*(*E*; ***β***), where

$$\begin{array}{c} \begin{array}{cc} f(E;\beta ) & =\int_{E_{0}}^{E}\;\exp(\int_{E_{0}}^{E^{\prime }}\;W\left(E^{\prime \prime };\beta \right)\;dE^{\prime \prime })\;dE^{\prime }\\ & =\sum_{i=1}^{[E]-1}\;\exp(\Delta \;\sum_{j=1}^{i-1}\;\beta_{j})\;\frac{\exp(\Delta \beta_{i})-1}{\beta_{i}}\\ & \;+\exp(\Delta \;\sum_{j=1}^{[E]-1}\;\beta_{j})\;\frac{\exp(\beta_{\left[E\right]}\left(E-\left(\left[E\right]-1\right)\Delta \right))-1}{\beta_{\left[E\right]}}. \end{array} \end{array}$$ (25)

We define [*E*] as the number of the bin that contains *E*. If *E* > *E*_1_, then we define [*E*] = *Q* + 1, and *β*_*Q*+1_ = 0.

Using this expression we can calculate the gradients ∇_***α***_ *F*(**s**; ***α***) in the algorithm exactly. This calculation is straightforward although the resulting expressions are cumbersome. For the semiparametric pairwise model, we have $F\left(s;\gamma ,\beta ,J\right)=V\;\left(\sum_{i,j=1}^{N}\;J_{ij}s_{i}s_{j};\gamma ,\beta \right)$. The gradient with respect to the couplings is

$$\begin{array}{c} \frac{\partial F(s;\gamma ,\beta ,J)}{\partial J_{kl}}=V^{\prime }\left(\sum_{i,j=1}^{N}\;J_{ij}s_{i}s_{j};\gamma ,\beta \right)s_{k}s_{l}. \end{array}$$ (26)

The gradients with respect to ***γ*** and ***β*** are just the gradients of *V* (*E*; ***γ***, ***β***) with respect to these parameters and they are as follows:

$$\begin{array}{c} \frac{\partial V\;(E;\gamma ,\beta )}{\partial \gamma_{1}}=1, \end{array}$$ (27)

$$\begin{array}{c} \frac{\partial V(E;\gamma ,\beta )}{\partial \gamma_{2}}=f\left(E;\beta \right), \end{array}$$ (28)

$$\begin{array}{c} \frac{\partial V(E;\gamma ,\beta )}{\partial \beta_{k}}=\gamma_{2}\frac{f(E;\beta )}{\partial \beta_{k}}. \end{array}$$ (29)

If *k* > [*E*], then

$$\begin{array}{c} \frac{\partial f(E;\beta )}{\partial \beta_{k}}=0. \end{array}$$ (30)

If *k* = [*E*], then

$$\begin{array}{c} \frac{\partial f(E;\beta )}{\partial \beta_{k}}=\exp\;(\Delta \;\sum_{j=1}^{[E]-1}\;\beta_{j})\;\frac{\exp\left(\Delta \beta_{\left[E\right]}\right)\Delta \beta_{\left[E\right]}-\exp\left(\Delta \beta_{\left[E\right]}\right)+1}{\beta_{\left[E\right]}^{2}}. \end{array}$$ (31)

If *k* < [*E*], then

$$\begin{array}{c} \begin{array}{cc} \frac{\partial f(E;\beta )}{\partial \beta_{k}} & =\exp\;(\Delta \;\sum_{j=1}^{k-1}\;\beta_{j})\;\frac{\exp\left(\Delta \beta_{k}\right)\Delta \beta_{k}-\exp\left(\Delta \beta_{k}\right)+1}{\beta_{k}^{2}}\\ & \;+\Delta \;\sum_{i=k+1}^{[E]-1}\;\exp\;(\Delta \;\sum_{j=1}^{i-1}\;\beta_{j})\;\frac{\exp(\Delta \beta_{i})-1}{\beta_{i}}\\ & \;+\Delta \exp\;(\Delta \;\sum_{j=1}^{[E]-1}\;\beta_{j})\;\frac{\exp\left(\beta_{\left[E\right]}\right)\left(E-\left(\left[E\right]-1\right)\Delta \right)-1}{\beta_{\left[E\right]}}. \end{array} \end{array}$$ (32)

### Estimating likelihoods

Data likelihoods cannot be evaluated exactly because the normalization constants *Z* are intractable. We resorted to Monte Carlo method known as thermodynamic integration in physics [54], and annealed importance sampling in machine learning, to estimate the normalization constants [55]. The initial model for annealed importance sampling was always the independent model for which the partition function can be calculated exactly. The sampling procedure consisted of 10^4^ intermediate distributions which uniformly interpolated from the independent model to the model of interest. Each partition function was estimated using 10^4^ samples.

All reported likelihoods were evaluated on held-out data. A simple cross-validation also showed that our models did not suffer from overfitting.

### Estimating the density of states and the latent variables

Density of states was estimated using the Wang and Landau algorithm [11, 40]. The accuracy parameter (the smallest increment size for the log of the density of states) was 10^−7^. The energy range was estimated during the first few thousand steps of the algorithm. This range was divided into ∼ 10^4^ bins. We decreased the increment size every ∼ 10^8^ iterations instead of checking energy histogram flatness since the later is hard to do when some energy bins are inaccessible.

We inferred the probability densities of the latent variables by considering the model in Eq (17) with fixed ***J*** which corresponds to the coupling matrix of the previously learned semiparametric pairwise model. The domain of the latent variable was set to [0, 5]. We approximated the integral with a sum by dividing this domain into 400 bins, and the value of the probability density *q*(*h*) was inferred by maximizing the likelihood of data subject to the constraint that *q*(*h*) integrates to 1. To make the computation tractable, we needed an expression for *Z*(*h*; ***J***). This can be obtained from the estimated density of states *ρ*(*E*; ***J***) of the energy as

$$\begin{array}{c} Z\left(h;J\right)=\sum_{s}\;e^{-hE(s;J)}=\int \;\rho \left(E;J\right)e^{-hE}\;dE. \end{array}$$ (33)
